# New Ointment Base

**Published:** 1916-03

**Authors:** 


**New Ointment Base.** 40 grams of petrolatum are mixed with 20 grams of powdered tragacanth, previously triturated with 30 grams of alcohol. 600 grams of milk whey, with whatever medicament may be desired, are added warm, little by little, with constant trituration. The whey is said to aid absorption. Chemiker Zeitung.

